# Dr. Walter H. Curtis

**Published:** 1917-05

**Authors:** 


**Dr. Walter H. Curtis,** Hahnemann of Philadelphia 1880, died at his home in Rochester Feb. 24, aged 56.

